# The diagnostic value of hepatobiliary scintigraphy for choledochal cysts in the era of magnetic resonance imaging with cholangiopancreatography and contrast-enhanced hepatobiliary phase: a case report and review

**DOI:** 10.1259/bjrcr.20210123

**Published:** 2022-03-09

**Authors:** Tak Kwong Chan, Wing Hang Luk, Fung Him Ng, Rois L.S. Chan, Yan Ho Hui, Chung Yan Justin Chan, Wai Hung Cheung

**Affiliations:** 1 Nuclear Medicine Unit, Queen Elizabeth Hospital, Yau Ma Tei, Hong Kong; 2 Department of Radiology, Princess Margaret Hospital, Yau Ma Tei, Hong Kong; 3 Department of Surgery, Princess Margaret Hospital, Yau Ma Tei, Hong Kong

## Abstract

**Objective:**

Choledochal cysts (CCs) represent cystic dilatations of the intra- or extrahepatic biliary tract. The diagnosis of CCs may not always be straightforward particularly for the intrahepatic subtype. Whereas the gold standard for diagnosing CCs is endoscopic retrograde cholangiopancreatography (ERCP), magnetic resonance cholangiopancreatography (MRCP) is commonly used as primary diagnostic tool for delineation of biliary pathologies including CCs.

**Methods:**

We report a case of cystic hepatic lesion near the confluence of bilateral intrahepatic ducts. MRCP shows direct anatomical communication between the lesion and the biliary tract, raising suspicion of a CC. Endoscopic ultrasound shows no communication between the lesion and biliary system. ^99m^Tc-hepatic iminodiacetic acid scintigraphy (hepatobiliary scintigraphy) was subsequently performed, showing no tracer uptake in the concerned cystic hepatic lesion despite visualisation of gallbladder and transit of tracer into the intestine. Overall scintigraphic findings speak against a CC.

**Conclusion:**

The case showed conflicting anatomical findings of a CC on MRCP and endoscopic ultrasound. Hepatobiliary scintigraphy and hepatobiliary contrast MRI may both functionally demonstrate communication of a hepatic lesion with the biliary tract. But hepatobiliary scintigraphy offers the advantage of much higher hepatic extraction and hence higher resistance to competition from plasma bilirubin compared with hepatobiliary contrast MRI. The better pharmacokinetics of HIDA confer superior lesion contrast that may offset inferior image spatial resolution, in particular for large lesions and patients with hyperbilirubinaemia. Hepatobiliary scintigraphy should be considered a suitable functional diagnostic modality for CCs even in the era of magnetic resonance imaging with cholangiopancreatography and contrast-enhanced hepatobiliary phase.

## Background

Choledochal cysts (CCs) represent cystic dilatations of the intra- or extrahepatic biliary tract. Approximately 80% of CCs are diagnosed within the first decade of life. The incidence of CCs ranges from 1 in 150,000 in the western world to 1 in 13,000 in Japan.^1^ CCs are four times more common in females. Anomalous pancreaticobiliary duct union (APBDU) is associated with 30 to 90% of CCs where the common bile duct joins pancreatic duct outside the duodenum, exposing biliary epithelium to pancreatic enzymes, which is postulated to contribute to the pathogenesis of CCs.^2^

The diagnosis of CCs may not always be straightforward particularly for the intrahepatic subtype. Around 10% of the population harbour one or more cystic lesions in the liver. The vast majority of non-parasitic cystic hepatic lesions being simple cysts, the differential diagnoses include degenerated tumours (primary or secondary), polycystic liver disease, and biliary cystic tumours and intrahepatic CCs. Whereas the gold standard for diagnosing CCs is endoscopic retrograde cholangiopancreatography (ERCP), ultrasound (US) and computed tomography (CT) allow detection of CCs by non-invasive means. The advent of magnetic resonance cholangiopancreatography (MRCP) has further improved the diagnostic tool for CCs. This said, a definitive diagnosis of intrahepatic CCs can still be difficult after exhausting these non-invasive radiological investigations. We present a case of suspected intrahepatic CC having undergone ultrasound, CT, MRCP and endoscopic ultrasound. The use of hepatobiliary scintigraphy excluded the diagnosis of an intrahepatic CC.

## Case

**Figure 1.**
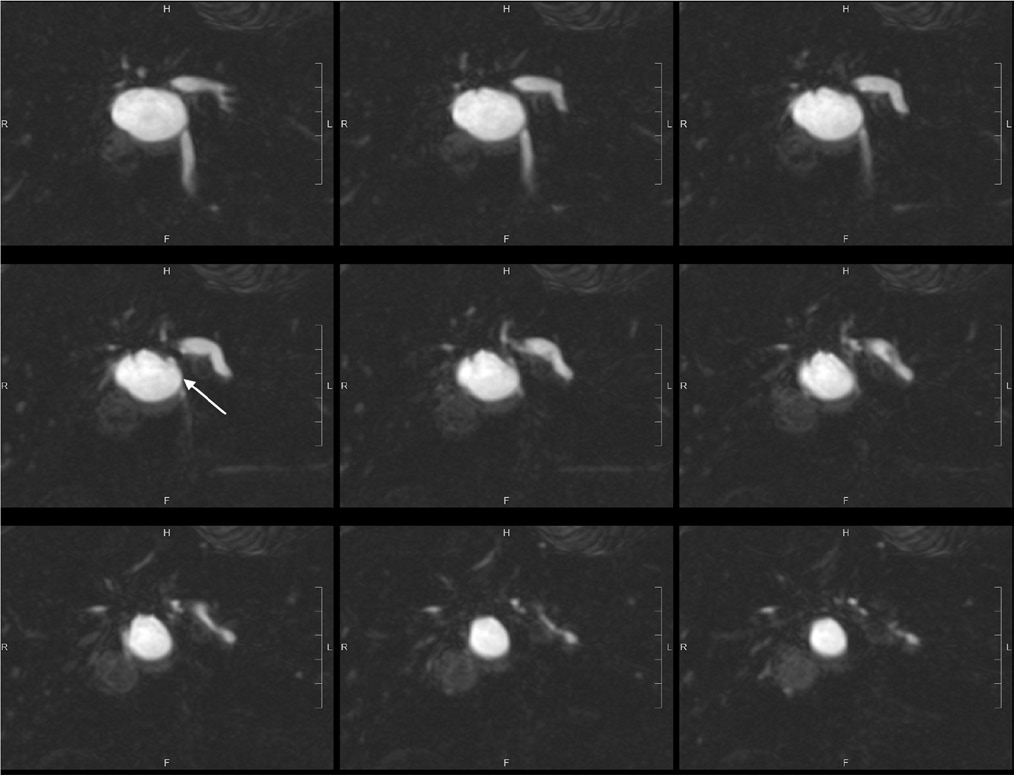
Post-oral contrast T2-weighted coronal MRCP images (SPACE 1mm with reformat) showed anatomical communication between the concerned cystic hepatic lesion near the confluence and right intrahepatic duct (white arrow).

**Figure 2.**
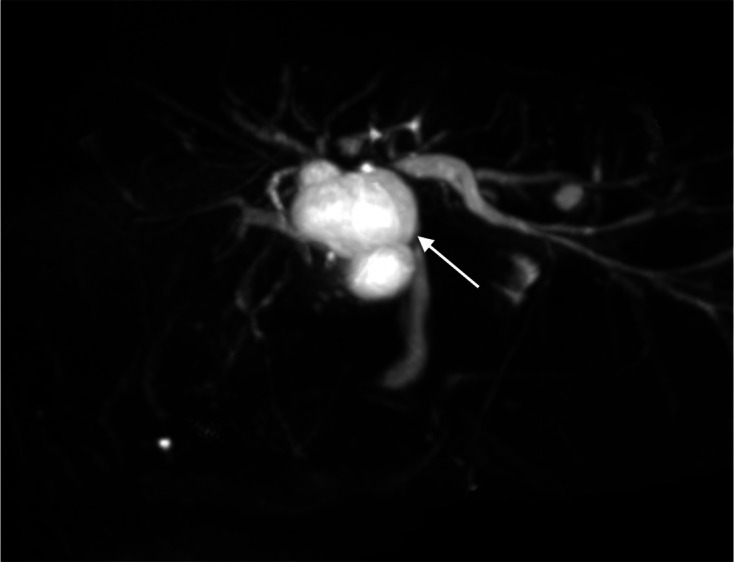
MRCP maximal intensity projection showed anatomical communication between the concerned cystic hepatic lesion near the confluence and right intrahepatic duct (white arrow).

**Figure 3.**
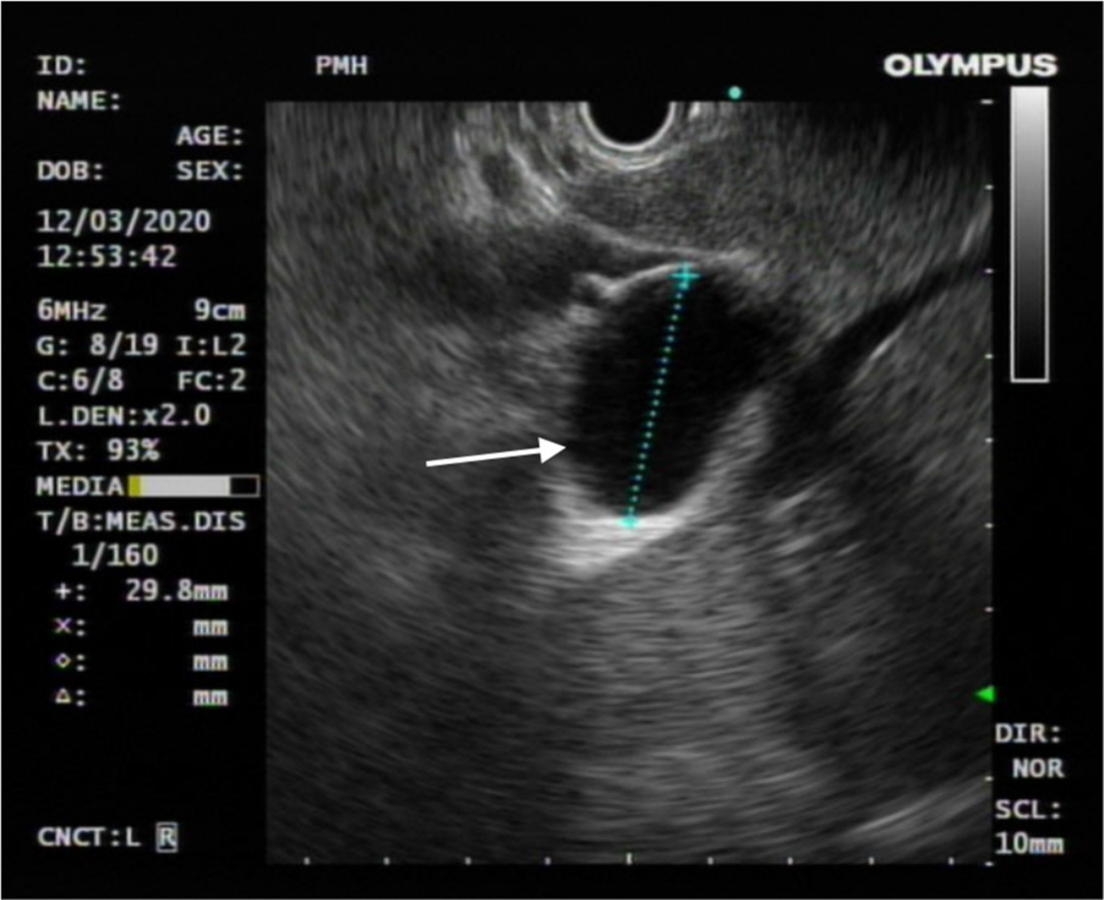
Endoscopic ultrasound showed no anatomical communication between the concerned cystic hepatic lesion and the biliary system (white arrow).

**Figure 4.**
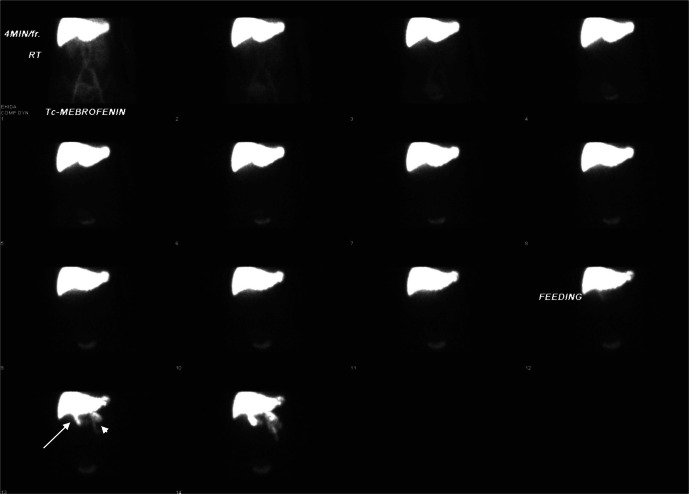
Hepatobiliary scintigraphy showed good hepatic tracer extraction with transit into gallbladder (white arrow) and intestine (white arrow head) during the first hour.

**Figure 5.**
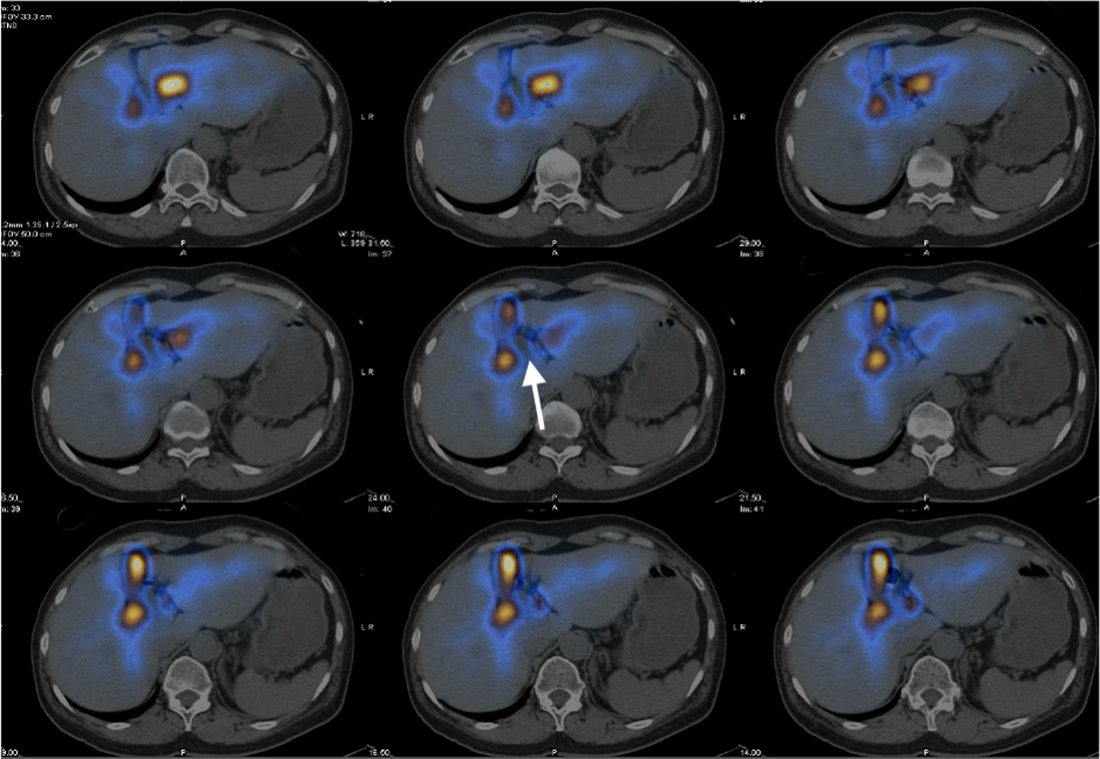
Hepatobiliary scintigraphy with SPECT/CT fusion images showed focal photopenia in the concerned cystic hepatic lesion (white arrow), suggestive of no communication with the biliary system.

**Figure 6.**
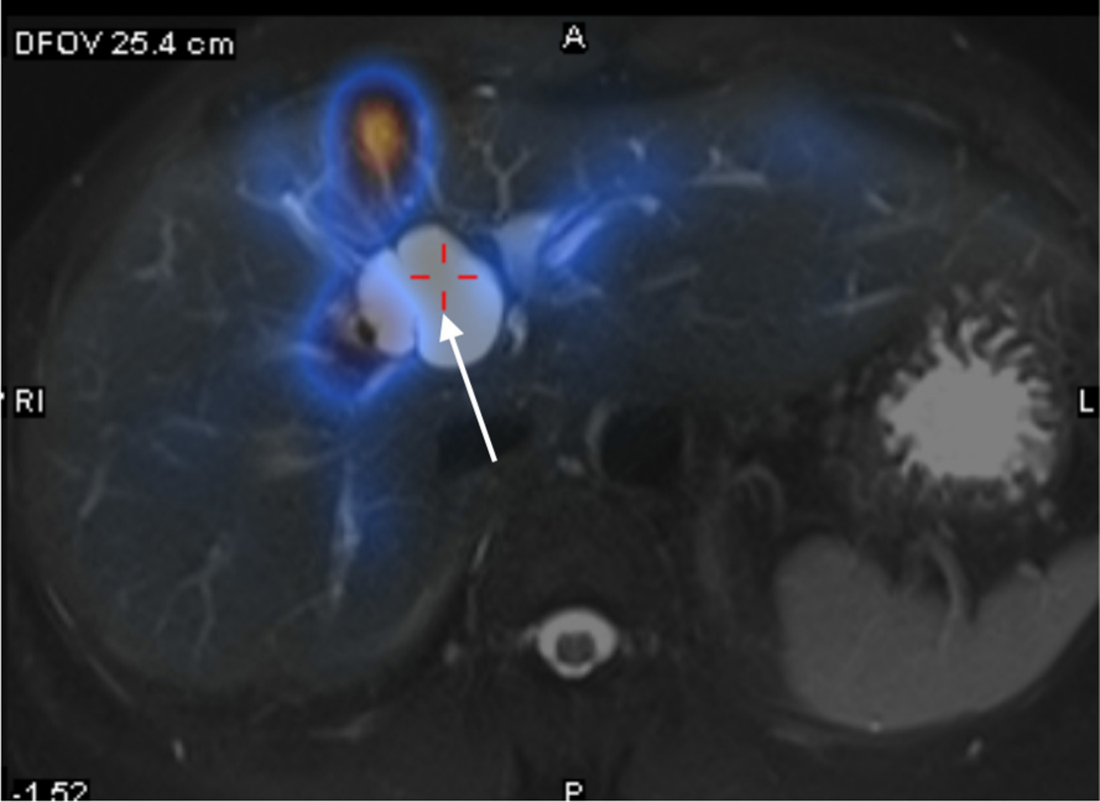
Manual co-registration of MRCP and SPECT images showed no tracer uptake in the concerned T2 hyperintense cystic hepatic lesion (white arrow).

The patient is a 57-year-old female presenting with abdominal distension. Ultrasound showed a 3.4-cm cystic hepatic lesion near the confluence. Subsequent CT confirmed the cystic lesion with internal septa with mildly dilated adjacent intrahepatic ducts. MRCP with no hepatobiliary contrast agent was then performed. The septated hepatic cyst was T1 hypointense and T2 hyperintense. *T_2_* weighted half-Fourier acquisition single-shot Turbo Spin Echo (HASTE) thick slice (50 mm) coronal images and reformatted *T_2_* weighted sampling perfection with application-optimised contrasts using different flip angle evolution (SPACE) thin slice (1 mm) coronal images were acquired following oral contrast, showing direct communication between the cystic lesion and extrahepatic portion of right intrahepatic duct (See Figures 1 and 2), raising suspicion of dilatation of the duct extending to proximal part of common bile duct. No APBDU was detected on MRCP. She then underwent endoscopic US which showed no communication between the concerned lesion and the biliary system (see Figure 3). Subsequent hepatobiliary scintigraphy showed normal transit of tracer activity into the biliary system and the intestine during the first hour of dynamic images (see Figure 4). Single-photon emission computed tomography (SPECT)/CT was also performed, showing photopenia in the concerned cystic hepatic lesion (See Figure 5). Manual co-registration of MRCP and SPECT images was also performed. The T2 hyperintense cystic hepatic lesion on MRCP showed no tracer uptake on the fused SPECT images (See Figure 6).

## Discussion

**Figure 7.**
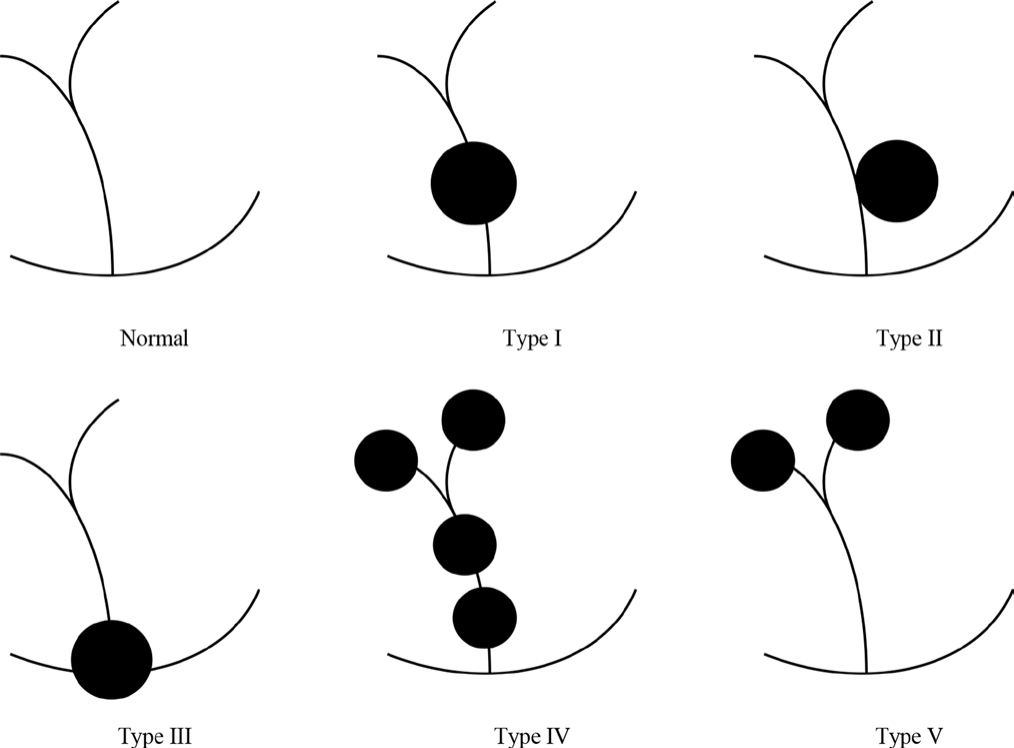
Todani classification of choledochal cysts. Type I are cystic dilation of the extrahepatic duct. Type II are juxtaposed to the common bile duct. Type III are characterised by their intraduodenal location at the pancreatic obiliary junction. Type IV are multiple lesions involving extrahepatic duct with or without intrahepatic involvement. Type V can be single or multiple intrahepatic saccular or fusiform dilatation.

The most widely accepted classification of CCs was devised by Todani and colleagues in 1977.^3^ Five types of CCs were described depending on the site of cystic change. Type I CCs typically involve extrahepatic biliary tract. Type II are juxtaposed to the common bile duct. Type III are characterised by their intraduodenal location at the pancreaticobiliary junction. Type IV are multiple lesions involving extrahepatic duct with or without intrahepatic involvement. Type V can be single or multiple intrahepatic saccular or fusiform dilatation (See Figure 7). A previous review showed that the frequencies of CCs using the Todani classification are as follows: type I (78%), type II (3%), type III (3%), type IV (15%) and type V (1%).^4^

While adults more likely present with biliary or pancreatic symptoms and abdominal pain, children commonly present with an abdominal mass and jaundice. Biliary malignancy is seen in 10 to 30% of adult CCs.^5–7^ The risk of cholangiocarcinoma in CC patients was reported to be more than 120 times that of the general population, and the risk remains high even after surgical excision of the CCs.^8,9^ Malignancy is most commonly associated with Types I and IV CCs, while types II, III, and V CCs have minimal risk.^10^ The cancer may arise either in the cyst wall itself, or in remnant tissue or any other part of the extrahepatic or intrahepatic bile duct.^11^ Due to the high risk of malignant transformation, Type 1 and Type IV extrahepatic CCs require complete resection, cholecystectomy and restoration of bilioenteric continuity.^12,13^ For Types II and III CCs, diverticlectomy, sphincterotomy or transduodenal excision may be indicated.^14,15^ With respect to Type IV intrahepatic CCs, some recommend partial hepatectomy but some prefer preservation unless the liver is cirrhotic. For Type V CCs, in the absence of cirrhosis or malignancy, Roux-en-Y cholangiojejunostomy with placement of stents may be indicated. Resection or liver transplant may be indicated in case of cirrhosis.^16^

Differential diagnoses of a CC include simple or haemorrhagic cysts, hydatid cysts, liver abscess, undifferentiated embryonal sarcoma, ciliated hepatic foregut cysts and cystic mesenchymal hamartoma. Other cystic lesions that can potentially communicate with the biliary tree include biliary cystadenoma, cystadenocarcinoma and the cystic variant of intraductal papillary neoplasm of the bile duct.^17,18^ Isolated saccular or fusiform CCs arising from the main intrahepatic bile ducts near the confluence have been reported. Whether these cysts represent true bile duct dilatation or other nature such as cystadenoma remains unknown.^19^

ERCP is the definitive diagnostic tool for evaluating CCs, but the procedure is associated with risks of serious complications. MRCP is commonly used as primary diagnostic tool for delineation of biliary pathologies including CCs. Type I to III CCs have more characteristic appearances and can usually be diagnosed on *T*_2_-weighted MRCP with confidence. On the other hand, Types IV and V CCs can be difficult to diagnose because they may appear as cystic collections with equivocal relationship to the biliary tree.^20^ A previous literature review showed that MRCP has an overall sensitivity of 96–100% for detection of CCs. The specificity was reported to be 90%.^21^ Biliary cystadenoma showing bile duct communication on MRCP and contrast-enhanced hepatobiliary phase magnetic resonance imaging (MRI) has been reported.^22^ This may be one of the examples of false-positivity for detection of CCs on MRCP.

Since 1970, hepatobiliary scintigraphy has been utilised to diagnose CCs. The most commonly used radiotracer nowadays is ^99m^Tc-hepatic iminodiacetic acid (HIDA) compounds. It has good imaging quality and allows functional assessment of the biliary tract even with bilirubin levels > 20 mg/dl.^23^ Hepatobiliary scintigraphy allows a functional diagnosis of CCs and can also define other anatomic or physiologic abnormalities of the hepatobiliary system. The classic finding of CCs on hepatobiliary scintigraphy is early photopenia with delayed fill-in (2 h).^24–26^ But actual findings can be variable: some may show early uptake within 1 h, while some never show any uptake due to biliary obstruction.

In our case, the concerned hepatic cyst showed no tracer uptake on delayed SPECT images up to about 2 h and upon co-registration of SPECT and MRCP images. A previous study demonstrated tracer uptake in all CCs within 1 h on hepatobiliary scintigraphy insofar as there is no delayed transit to the intestine.^27^ As the patient shows early visualisation of the gallbladder and transit of the tracer activity into the intestine, the absence of tracer activity in the concerned cyst cannot be attributed to delayed transit or obstruction. Overall scintigraphic findings in our case can confidently exclude a CC.

Alongside hepatobiliary scintigraphy, hepatobiliary contrast-enhanced *T*_1_-weighted MRI may also functionally demonstrate communication between a cystic hepatic lesion and the biliary tree. There are two gadolinium-based liver-specific compounds gadoxetic acid (Primovist^®^, Bayer Schering Pharma, Berlin, Germany) and gadobenate dimeglumine (MultiHance^®^, Bracco Imaging, Milan, Italy) approved for use by Food and Drug Administration of the United States. Primovist^®^ shows more favourable contrast pharmacokinetics. Still only up to 50% of the contrast is extracted in the liver and the bile duct to liver contrast ratio is only up to 0.7 for delayed imaging up to 300 min following administration of contrast ^26^.

Compared with MRI with contrast-enhanced hepatobiliary phase, hepatobiliary scintigraphy offers the advantage of much higher hepatic extraction (98% for mebrofenin; 50% for Primovist^®^) and thereby higher resistance to competition from plasma bilirubin.^23^ Although no head-to-head comparison is available, the better pharmacokinetics of HIDA possibly confers a superior lesion contrast that may offset the inferior image spatial resolution compared with MRI, in particular for large lesions and in patients with hyperbilirubinaemia. Further, hepatobiliary contrast has to be used with caution in neonates or patients with impaired renal function.^27^

## Conclusion

In summary, we report a case of conflicting anatomical findings of a CC on MRCP and endoscopic ultrasound. When a functional study is required to further delineate communication between a cystic lesion and the biliary system, hepatobiliary scintigraphy should be considered a suitable diagnostic modality, even in the era of magnetic resonance imaging with cholangiopancreatography and contrast-enhanced hepatobiliary phase.

## Learning points

1. MRCP has an overall sensitivity of 96–100% and specificity of 90% for detection of CCs.
2. Hepatobiliary scintigraphy and hepatobiliary contrast MRI may both functionally demonstrate communication of a hepatic lesion with the biliary tract.
3. Hepatobiliary scintigraphy offers the advantage of much higher hepatic extraction and hence higher resistance to competition from plasma bilirubin compared with hepatobiliary contrast MRI. The better pharmacokinetics of HIDA confer superior lesion contrast that may offset inferior image spatial resolution, in particular for large lesions and patients with hyperbilirubinaemia.
4. When a functional study is required to further delineate communication between a cystic lesion and the biliary system, hepatobiliary scintigraphy should be considered a suitable diagnostic modality.
